# Neural Oscillatory and Network Signatures of Age-Related Cognitive Decline Under Motor-Cognitive Dual-Task Conditions

**DOI:** 10.3390/brainsci16030335

**Published:** 2026-03-21

**Authors:** Miaomiao Guo, Qi Wang, Mengfan Li, Liang Sun, Tian Wang, Guizhi Xu, Lei Wang

**Affiliations:** 1 State Key Laboratory of Intelligent Power Distribution Equipment and System, School of Health Sciences and Biomedical Engineering, Hebei University of Technology, Tianjin 300130, China; gmm@hebut.edu.cn (M.G.); 202322902004@stu.hebut.edu.cn (Q.W.); mfli@hebut.edu.cn (M.L.); 202312901004@stu.hebut.edu.cn (T.W.); gzxu@hebut.edu.cn (G.X.); 2 Tianjin Key Laboratory of Bioelectromagnetic Technology and Intelligent Health, School of Health Sciences and Biomedical Engineering, Hebei University of Technology, Tianjin 300130, China; 3 The NHC Key Laboratory of Geriatrics, Institute of Geriatric Medicine, Chinese Academy of Medical Sciences, Beijing Hospital/National Center of Gerontology of National Health Commission, Beijing 100730, China; sunliang3860@bjhmoh.cn; 4 Institute of Biomedical Engineering, Chinese Academy of Medical Sciences & Peking Union Medical College, Tianjin 300192, China; 5 State Key Laboratory of Advanced Medical Materials and Devices, Tianjin 301600, China

**Keywords:** brain aging, motor-cognitive dual-task, neural oscillations, functional connectivity, multimodal

## Abstract

**Background**: Against the backdrop of global population aging, understanding the mechanisms of age-related cognitive decline has become crucial for improving the health and quality of life in older adults. **Methods**: This study employed a multimodal approach to investigate the neural modulations induced by a motor cognitive dual task and their relationship with age-related decline. By integrating behavioral assessments, electroencephalography (EEG), and body composition analysis, we comprehensively evaluated performance and neural correlates in 19 younger and 18 older adults. Specifically, EEG analyses focused on comparing pre-task and post-task resting-state recordings to investigate the immediate impact of a single acute cognitive-motor dual-task session on neural oscillations and brain network organization. **Results**: Key findings include: (1) older adults exhibited significantly inferior performance in task accuracy, reaction time, and composite performance score compared to younger adults (*p* < 0.001); (2) neural oscillatory analysis of resting-state data revealed a localized increase in gamma-band power at posterior-temporal sites (PO4/T6) in older adults following the dual-task, while younger adults exhibited widespread multi-band (delta to beta) power modulation across frontal, central, and temporal regions in younger adults; (3) brain network analysis demonstrated synergistic enhancement of multi-band (Theta, Alpha, Beta, Gamma) connectivity and optimized topological organization in younger adults post-task, contrasting with network rigidity and localized compensatory patterns in older adults; (4) correlation analyses indicated significant associations between dual-task performance and MoCA-B scores in older adults (r = 0.861, *p* < 0.001). **Conclusions**: This study innovatively elucidates the neurophysiological characteristics of brain aging. The motor-cognitive dual-task paradigm proves to be a sensitive tool for capturing early cognitive changes, holding significant promise for clinical screening.

## 1. Introduction

With the accelerating global trend of population aging, cognitive decline has emerged as a significant challenge impacting the health and quality of life among older adults. Cognitive impairment impairs independent living and imposes a substantial caregiving and socioeconomic burden on families and society. Therefore, developing efficient and objective methods for early screening is a critical priority for aging societies.

Current approaches to early cognitive screening primarily rely on neuropsychological scales such as the Mini-Mental State Examination (MMSE) and Montreal Cognitive Assessment (MoCA), while the diagnosis of brain aging predominantly utilizes neuroimaging techniques, including functional magnetic resonance imaging (fMRI) and positron emission tomography (PET) [1]. However, traditional scales are vulnerable to subjective biases and exhibit limited sensitivity to subtle cognitive changes. Although fMRI and PET provide high spatial resolution for assessing brain structure and metabolic activity, their prohibitive costs and complex operational requirements severely restrict widespread adoption in primary healthcare settings.

To address these limitations, recent research has explored alternative methodologies. The dual-task (DT) paradigm, for instance, has gained considerable attention for its capacity to simulate complex cognitive scenarios and effectively evaluate attentional resource allocation and executive functions [2]. Complementing this approach, electroencephalography (EEG), a non-invasive neurophysiological technique with high temporal resolution, enables precise capture of dynamic neural oscillations and functional network connectivity patterns. By integrating DT paradigms with EEG technology, researchers can achieve a comprehensive characterization of cognitive aging across behavioral performance, neural oscillations, and functional networks. Emerging evidence indicates that motor-cognitive dual-task training induces measurable neuroplastic changes. Studies show it increases neural oscillation frequency [3] and theta synchronization [4], while EEG findings reveal stronger P300 responses and altered spectral power, particularly in frontoparietal regions of older adults [5,6]. Neuroimaging evidence indicates prefrontal and sensorimotor activation, along with improved brain network efficiency during dual-tasking [7,8]. Furthermore, accumulating evidence suggests that dysregulation in dynamic neural oscillations may serve as a key neural marker of cognitive decline [9,10,11].

Beyond neural metrics, metabolic factors have also been implicated in cognitive aging. Extensive epidemiological studies demonstrate significant associations between obesity and elevated risk of cognitive deterioration [12,13], as well as strong correlations between musculoskeletal dysfunction and cognitive impairment [14,15]. Despite these advances, critical gaps persist in current research. Most studies focus on isolated neural metrics like behavioral performance or narrow-band oscillations, rather than conducting integrated multimodal analyses. Furthermore, the clinical utility of dual-task paradigms for cognitive screening remains under-validated. Most importantly, the tripartite interactions among metabolic regulation, neural electrophysiological dynamics, and cognitive function remain poorly understood, particularly in the context of motor-cognitive integration.

To address these gaps, our approach leverages combined EEG and body composition analysis to provide a comprehensive assessment. We aim to: (1) systematically compare behavioral performance in DT and baseline metabolic differences between younger and older adult groups; (2) elucidate age-related effects on neural oscillation modulation and functional network reorganization; and (3) establish the relationships among metabolic indices, neural oscillations, and cognitive function, validating the advantages and feasibility of DT paradigms in cognitive assessment. Our study is expected to offer novel insights into brain aging mechanisms, ultimately providing an objective approach for early cognitive decline screening.

## 2. Materials and Methods

### 2.1. Participants

A total of 37 participants were enrolled in this study, including 18 older adults (aged 55–70 years) and 19 younger adults (aged 18–30 years). Exclusion criteria encompassed: (1) history of neurological/psychiatric disorders; (2) sensory, cognitive, or motor impairments affecting task performance; and (3) postoperative recovery from major surgery, pregnancy, or other health conditions contraindicated for physical exertion. All participants were screened for basic motor function to confirm their ability to tolerate moderate-intensity exercise for at least 15 min, and all successfully completed resting-state EEG recordings. The study was approved by the Ethics Committee of the School of Health Sciences and Biomedical Engineering, Hebei University of Technology (No. HEBUThMEC2023021), with written informed consent obtained from all participants. All participants received financial compensation for their participation.

### 2.2. Experimental Design

**Figure 1:**
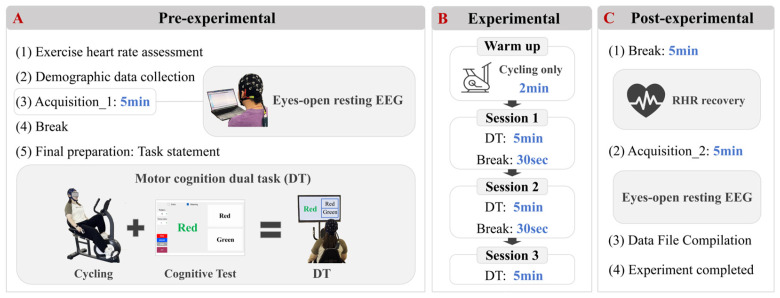
Experimental protocol. The experiment consisted of three phases. (**A3**) Under Phase I illustrates the schematic of resting-state EEG signal acquisition, while (**A5**) displays the diagrams of cycling and cognitive testing, respectively. (**B**) Provides a detailed description of each session in the DT experiment. (**C**) Illustrates the specifics of the post-experiment phase.

The study comprised three consecutive phases: pre-experimental, experimental, and post-experimental (Figure 1). During the pre-experimental phase, all participants received comprehensive instruction on experimental procedures from the research team. Each participant underwent exercise heart rate assessments, involving measurement of resting heart rate (RHR) after 10 min of seated rest, estimation of individual maximum heart rate [16], and calculation of moderate-intensity target heart rate ranges [17]. Baseline characteristics (Baseline refers to the pre-task phase, prior to the execution of both single and dual tasks.) including gender, age, education years, height, body mass index (BMI), skeletal muscle mass index (SMI), lower limb extensor peak torque assessed via knee isokinetic dynamometry, and visceral fat area (VFA) were recorded. BMI, SMI, and VFA measurements were acquired via bioelectrical impedance analysis (BIA). Participants then completed a 5 min open-eye resting-state EEG recording while seated (Pre-task Rest), followed by a 5 min rest period.

During the experimental phase, the stationary bicycle resistance was set to a constant load of 70w. Continuous heart rate monitoring during experimental sessions was implemented using a Masimo portable pulse oximetry device (Masimo Corporation, Irvine, CA 92618, USA). Participants initially cycled for 2 min to elevate their heart rate into the moderate-intensity zone. Subsequently, they performed three 5 min blocks of a mixed-mode Stroop task during continuous moderate-intensity cycling. The Stroop task was performed using an intelligent cognitive assessment system (Tianjin Ruiqi Medical Technology Co., Ltd., Tianjin, China, with version number 2026SR0115945V1.0). The paradigm required participants to vocally respond to either font colors or word meanings as prompted by the digital interface. Stimuli representing font colors and word meanings were presented with equal probability using a response-contingent progression: upon vocal response detection by the system, the subsequent stimulus was immediately displayed, regardless of answer accuracy. Although a nominal inter-stimulus interval of 3 s was set as a maximum threshold, the actual presentation rate depended on individual reaction times, resulting in a variable number of trials across participants. To mitigate fatigue, 30 s rest intervals separated each task block. Task performance metrics (completed items, correct responses, accuracy rate, mean reaction time) were automatically recorded. For the behavioral analysis, the optimal performance indices (e.g., highest accuracy or fastest reaction time) across the three blocks were selected for each participant to represent their peak cognitive-motor capability. Researchers continuously monitored heart rates, instructing cadence adjustments (increasing or decreasing) to maintain target intensity throughout the task blocks.

In the post-experimental phase, participants rested for approximately 5 min until returning to resting-state heart rate levels, followed by a final 5 min open-eye resting-state EEG recording session (Post-task Rest).

Although EEG signals were recorded during these active tasks, they were excluded from primary analyses due to substantial motion and vocalization artifacts. The study focused on task-induced neural after-effects by comparing resting-state activity before and after the DT. Consequently, all reported spectral, connectivity, and graph-theoretical analyses were conducted exclusively on the Pre-task and Post-task resting-state segments to assess modulations in neural activity and network organization relative to the baseline.

### 2.3. EEG Data Acquisition, Processing and Analysis

#### 2.3.1. Acquisition

Resting-state EEG signals were acquired using a 32-channel wireless EEG system (NeuSen W132, Neuracle Technology (Changzhou) Co., Ltd., Changzhou, China) within an acoustically shielded chamber (ambient noise < 40 dB). Participants maintained a comfortable seated position throughout. Electrodes were positioned according to the International 10–20 system, including bilateral mastoid electrodes (A1, A2), one ground electrode (GND), and one physical reference electrode (REF). Scalp contact sites were degreased using medical alcohol before conductive gel application to ensure stable electrode-skin impedance below 5 kΩ; particular attention was paid to the GND and REF electrodes to guarantee hardware stability and reliable impedance monitoring during setup. The sampling rate was set to 1000 Hz. While the acquisition software displayed an online average reference for real-time monitoring, the raw data were recorded relative to the physical REF electrode.

#### 2.3.2. Preprocessing

Raw EEG data were preprocessed systematically using the EEGLAB toolbox (version 23.1) within the MATLAB environment [18]. To prevent signal contamination and ensure methodological consistency across all participants, bilateral mastoid electrodes (A1, A2) were uniformly removed at the initial stage. Subsequently, data were subjected to bandpass filtering (0.1–45 Hz) followed by a 50 Hz notch filter to eliminate residual line noise. Data were then downsampled to 500 Hz. For artifact removal, Independent Component Analysis (ICA) was performed on the high-pass filtered (0.1 Hz) continuous data using the Infomax algorithm (runica.m, default settings). Physiological artifacts identified via semi-automated inspection and manual validation were removed. Anomalous epochs were excluded from the cleaned data before finally rereferencing to the whole-brain average calculated from the remaining clean scalp electrodes, thereby minimizing spatial bias associated with the physical reference while maintaining a uniform analytical framework.

#### 2.3.3. Power Spectral Density

To compare neural oscillations across age groups during DT, spectral features were extracted using Welch’s method across five specific frequency bands [19]: Delta (0.5–4 Hz), Theta (4–8 Hz), Alpha (8–12 Hz), Beta (12–30 Hz), and Gamma (30–45 Hz). Data were segmented into 5 s epochs with a Hamming window. To maximize data utilization, a sliding step of 1 s was applied, resulting in an overlap of 2000 samples (80%). Given a sampling rate of 500 Hz, each segment contained 2500 samples. We set the FFT length (nfft) to 2500, yielding a final spectral frequency resolution of 0.2 Hz. Absolute power spectral density (PSD) was computed per channel and averaged across epochs. All analyses were implemented using custom scripts written in MATLAB.

#### 2.3.4. Brain Functional Networks

The weighted phase lag index (wPLI) quantifies functional connectivity by assessing phase lag stability while minimizing volume conduction effects [20]. Its noise robustness and suitability for brief EEG segments make it ideal for dynamic network analysis. We computed wPLI networks using a sliding window approach: segmenting EEG into 2 s epochs; mapping 30 electrodes to 16 regions (see Figure S1 and Table S1) using a non-weighted simple average; extracting five frequency bands (Delta to Gamma); deriving phase and amplitude via Hilbert transform; computing cross-spectra imaginary components; calculating wPLI per epoch (Equation (1)); and averaging across windows to generate 16 × 16 connectivity matrices. The MATLAB script used to calculate the weighted wPLI, named wpli_compute.m, is provided in the Supplementary Materials. It is important to note that “connectivity strength” in the following text was defined as the weight coefficient between any two brain regions in the functional connectivity matrix constructed based on the wPLI.

(1) wPLI=EImCEImC,

Here, *C* represents the cross-spectrum.

To comprehensively quantify functional connectivity differences between age groups, six graph theory metrics were computed from wPLI matrices to assess age-related connectivity differences: global efficiency (Eg), local efficiency (Eloc), characteristic path length (Lp), clustering coefficient (Cp), degree centrality (Dc), and betweenness centrality (Bc). Using GRETNA [21], we analyzed topological attributes across sparsity thresholds (0.2–0.6, stepsize = 0.05), with area-under-curve values calculated to ensure threshold-independent results. All analyses were implemented using custom scripts written in MATLAB.

#### 2.3.5. Participant Characteristics and Behavioral Metrics

Demographic data (gender, age, education, BMI, SMI, VFA, lower limb extensor peak torque(P_LM)) and task performance metrics (accuracy, time) were recorded. Additionally, traditional cognitive assessments were conducted using the Montreal Cognitive Assessment-Beijing version (MoCA-B), which was administered exclusively to the older adult group. A Performance Index (Perf, see Equation (2)) combining response count and speed was derived to holistically assess dual-task performance, mitigating ceiling effects in high-performing groups by capturing speed–accuracy tradeoffs.

(2) $$Perf=\frac{N_{C}}{T},$$

where *N_c_* is the number of correct questions answered during the task and *T* is the average response time.

Additionally, univariate linear regression analyses were conducted to examine: (1) correlations between traditional cognitive assessments and DT performance; (2) associations between dual-task-induced power spectral density changes and performance metrics; (3) relationships linking traditional cognitive scores and spectral alterations with adipose and muscular metrics.

#### 2.3.6. Statistical Analysis

Normality was assessed using the Shapiro–Wilk test [22]. Independent samples *t*-tests compared intergroup differences in demographics and task performance for normally distributed data, reporting *Cohen’s d* as the effect size; the Mann–Whitney U test was applied for non-normal distributions, reporting Cliff’s d [23]. Within-group pre-post changes in PSD and network connectivity were analyzed using paired samples *t*-tests (normal distributions) or Wilcoxon signed-rank tests (non-normal distributions), reporting estimated effect size in nonparametric power analysis ($\hat{p}$) [24]. To address multiple comparisons, we applied two correction approaches. Network-based statistic (NBS): For connectivity analyses, NBS (threshold: *p* < 0.01, cluster-forming) was used to correct for topological dependencies [25], with family-wise error rate controlled via 5000 permutations using the GRETNA toolbox (implemented in MATLAB R2020b). False discovery rate (FDR) correction: The *p*-values were adjusted using the Benjamini–Hochberg correction method [26], and we denoted the corrected *p*-values as *p*(*fdr*). Furthermore, for the regression analyses, we generated confidence intervals (95% CI) using bias correction and accelerated bootstrap methods based on 2000 resamples. All conventional statistical analyses were performed using IBM SPSS Statistics version 26.0. A significance level of 0.05 was set for all statistical analyses.

## 3. Results

### 3.1. Demographic Data

Baseline characteristics including sex, age, education duration, MoCA-B scores, height, BMI, SMI, VFA and P_LM are presented in Table 1 for both groups (Group O: older adults; Group Y: younger adults). Statistical comparisons revealed that the two groups differed significantly in age and years of education (*p* < 0.001). However, no significant differences were observed in other demographic parameters. The data are presented as Mean ± Standard Deviation.

### 3.2. Task Performance

**Figure 2:**
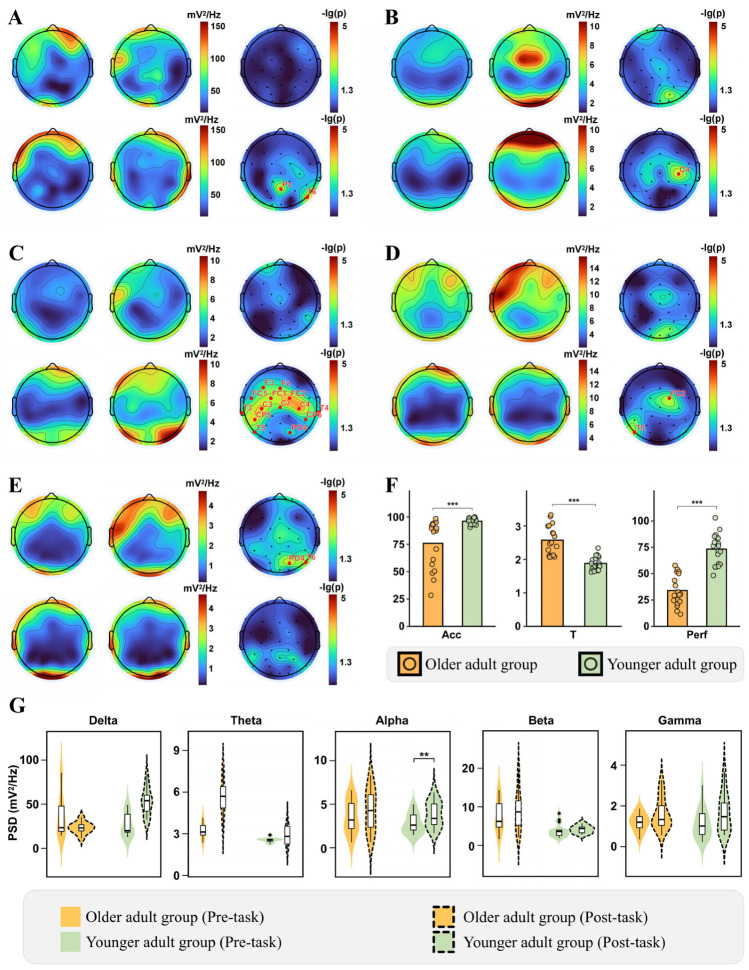
PSD changes and task performance under dual-task conditions across age groups. (**A**–**E**) Display topographical PSD distributions (Delta to Gamma bands). Each subpanel comprises six topoplots: the first row (second row) represents the older adults group (younger adults group), while the first column (second column) denotes the pre-task (post-task) state. The third column highlights electrode locations and names with statistically significant PSD changes (corrected for multiple comparisons) during dual-task performance. (**F**) Quantifies task performance (Acc, T, and Perf) between the two groups. (**G**) Illustrates the whole-brain averaged PSD levels for Delta, Theta, Alpha, Beta, and Gamma bands (left to right). ** *p* < 0.01, *** *p* < 0.001.

After all participant groups completed the motor-cognitive dual-task paradigm, we recorded their cognitive task performance during dual-task execution, including the following metrics: accuracy rate (Acc), mean response time (T), and Perf. Statistical analyses of task performance across age groups revealed non-normal distribution for Acc, necessitating Mann–Whitney U tests, while independent samples *t*-tests were applied to T and Perf. As shown in Figure 2F, younger participants demonstrated significantly higher Acc and Perf during DT performance versus older adults (Acc: *U* = 305, *p* < 0.001, *Cliff’s d* = 0.784, *power* = 87.2%; Perf: *t* = 8.255, *p* < 0.001, *Cohen’s d* = 2.791, *power* = 99.9%), whereas older adults exhibited significantly longer response times (T: *t* = −6.244, *p* < 0.001, *Cohen’s d* = 2.054, *power* = 99.8%).

### 3.3. Power Spectral Density

To compare neural oscillatory signatures across age groups under dual-task conditions, we analyzed changes in PSD across frequency bands before and after DT performance in each group. After completing the DT, older adults exhibited a general trend of increased power spectral density (PSD) across all frequency bands (Figure 2A–E), with only the Gamma band showing a significant enhancement at the PO4 and T6 electrodes after FDR correction (both *p*(*fdr*) = 0.030).

In contrast, young adults showed a significant increase in PSD across several isolated electrodes in the Delta, Theta, and Beta frequency bands. Most noteworthy is that the strongest result in pre- post task differences is obtained in young adults in the alpha band in the bilateral central-parietal-temporal axis (statistical analysis results are shown in Table 2).

### 3.4. Functional Connectivity

**Figure 3:**
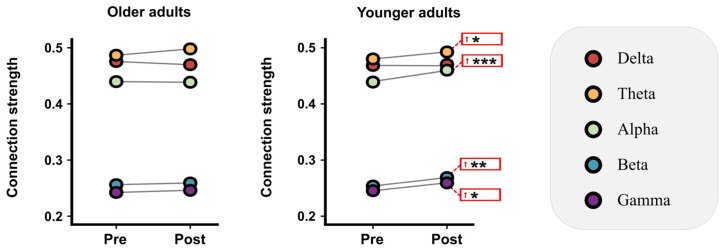
Age-related alterations in functional brain networks during dual-task performance: Whole-brain connectivity strength. Whole-brain connectivity strength changes (arrows indicate post-task enhancement). * *p* < 0.05, ** *p* < 0.01, *** *p* < 0.001.

**Figure 4:**
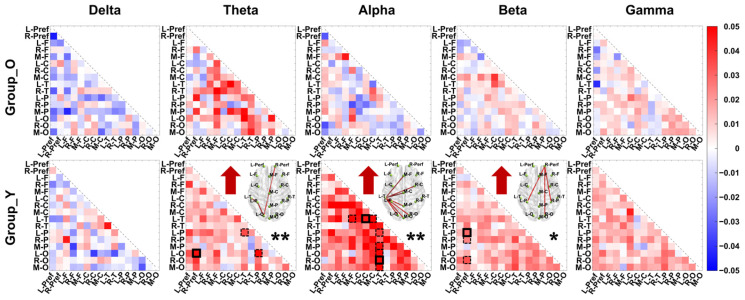
Age-related changes in functional brain networks during dual-task performance: Alterations in interregional connectivity strength. Differences in pairwise nodal connectivity strength during dual-task performance, defined as the post-DT wPLI matrix minus the pre-DT wPLI matrix, highlighting significant trans-hemispheric (the black solid boxes) and non-trans-hemispheric (the black dashed boxes) connectivity changes. Upper triangular arrows indicate post-DT subnetwork enhancement or weakening versus pre-DT. The upper triangle shows the nodes and edges of the significantly enhanced subnetwork after the DT. * *p* < 0.05, ** *p* < 0.01.

This study investigated changes in frequency-specific functional network connectivity strength pre-to-post-DT across groups. Firstly, the mean values of the connection strength between all brain regions in the functional network were analyzed. Older adults showed no significant connectivity changes across bands (see Figure 3). Younger adults exhibited significant whole-brain connectivity increases in Theta (*t* = 2.485, *p* = 0.023, *d* = 0.570, *power* = 65.2%), Alpha (*t* = 4.186, *p* < 0.001, *d* = 0.960, *power* = 97.7%), Beta (*t* = 3.229, *p* = 0.005, *d* = 0.741, *power* = 86.3%), and Gamma (*t* = 2.691, *p* = 0.015, *d* = 0.617, *power* = 72.1%) bands (statistical analysis results are shown in Table S2). Further, connection strength changes (pre-to-post DT) were analyzed for each age group, with NBS identifying significantly altered subnetworks. NBS analysis revealed no significantly altered subnetworks in older adults. However, young participants demonstrated significantly enhanced subnetworks in multiple frequency bands (Theta, Alpha, and Beta) following the same task conditions (Theta: *t* = 3.465, *p* = 0.008, *Cohen’s d* = 0.795, *power* = 90.6%; Alpha: *t* = 4.174, *p* = 0.002, *Cohen’s d* = 0.958, *power* = 97.6%; Beta: *t* = 3.380, *p* = 0.033, *Cohen’s d* = 0.775, *power* = 89.2%). Notably, within the alpha and beta frequency bands, the left temporal lobe and right prefrontal lobe served as central nodes in their respective subnetworks, acting as network hubs. In detail, the subnetwork with significantly enhanced connectivity in the Alpha band is formed by the connections between the L-T (left temporal) region and the M-F, R-C, M-C, M-P, R-O, M-O, and L-O regions. In the Beta band, the subnetwork with significantly enhanced connectivity in the past is formed by the connections between the R-Perf (right perfrontal) region and the L-P, R-O, and R-P regions. These findings are detailed in Figure 4.

**Figure 5:**
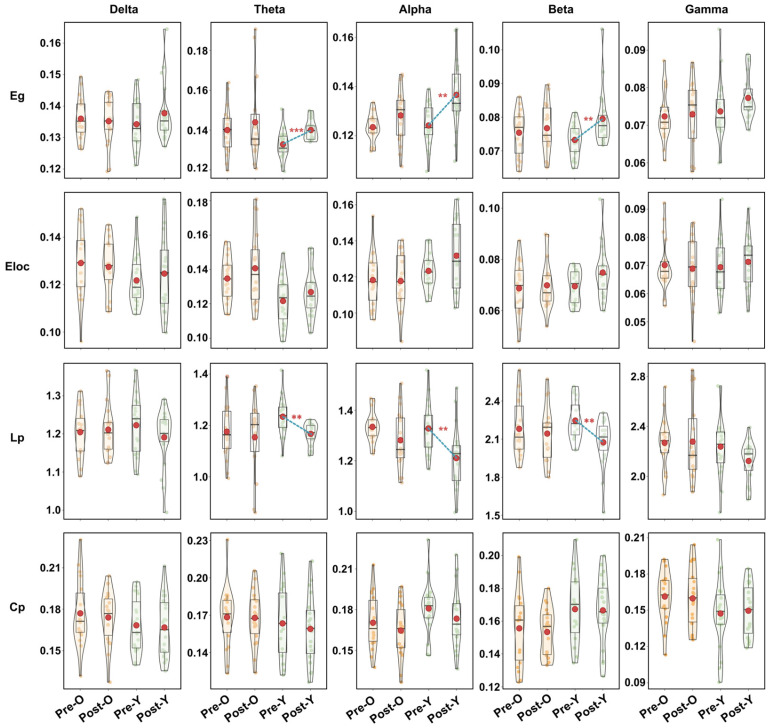
Age-related alterations in functional brain networks during dual-task performance: global topological properties. Global topology changes pre-to-post DT: red dots show metric means, dotted lines mark significant change trends. ** *p* < 0.01, *** *p* < 0.001.

**Figure 6:**
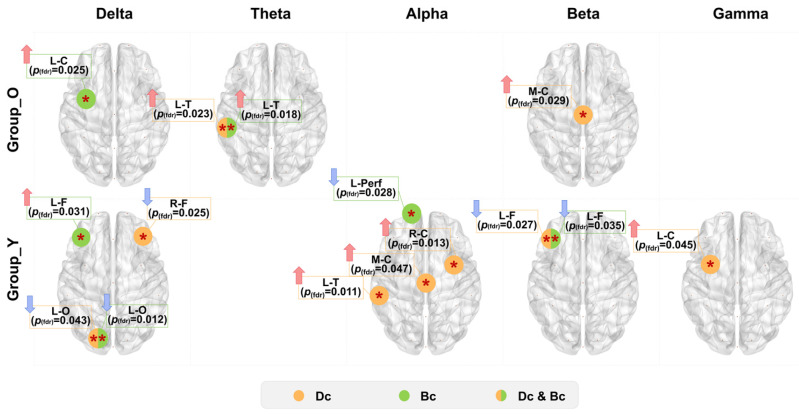
Age-related changes in functional brain networks during dual-task performance: Alterations in local topological properties. Pre-to-post-task changes in local topological properties (Dc and Bc), with directional arrows denoting index trends. * *p* < 0.05, ** *p* < 0.01.

For network topology analysis, global properties (Eg, Eloc, Lp, Cp) per frequency band were computed and visualized via box-violin plots. Nodal metrics (Dc, Bc) were analyzed to assess DT effects. Older adults exhibited no significant global metric changes post-task. Younger adults demonstrated significantly increased Eg and decreased Lp in Theta, Alpha, and Beta bands (Figure 5). For nodal metrics, older adults showed significant Dc increases in left temporal region (Theta) and central region (Beta), with Bc increases in left central region (Delta) and left temporal region (Theta). Younger adults had complex nodal changes: Dc decreased in right frontal and left occipital regions (Delta), increased in central and left temporal regions (Alpha) and left central region (Gamma), and decreased in left frontal region (Beta); Bc increased in left frontal region but decreased in left occipital region (Delta), left prefrontal (Alpha) and left frontal region (Beta), see Figure 6. The detailed results of the above statistical analysis are shown in Tables S4 and S5.

### 3.5. Correlation Analysis

**Figure 7:**
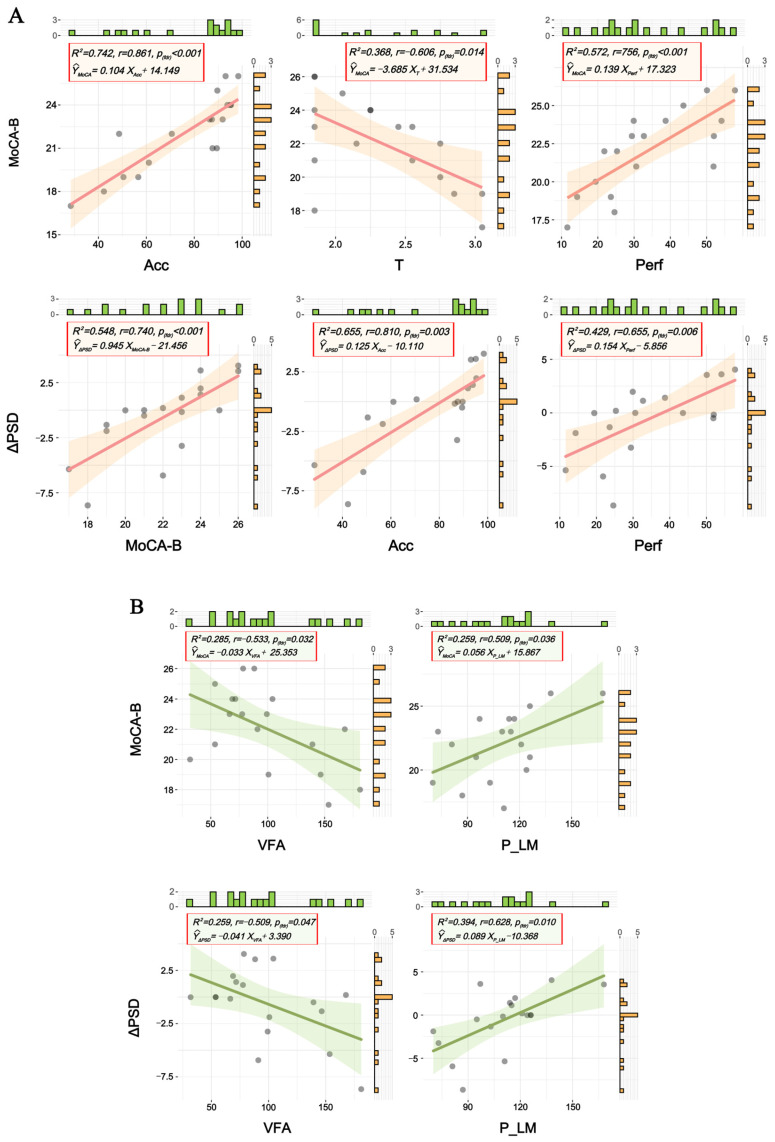
Univariate linear regression analysis of multimodal data. (**A**) Statistical association: DT performance and MoCA-B score, DT performance and ΔPSD. (**B**) Statistical association: metabolic indicators and MoCA-B score, metabolic indicators and ΔPSD. Marginal histograms are displayed along each axis to illustrate the distribution of the respective variables. The dots in the figure represent sample points.

This study employed simple linear regression to examine the associations between cognitive function (as measured by MoCA-B scores in older adults), DT performance metrics (Acc, T and Perf), metabolic indicators (VFA, P_LM), and neural oscillation changes (ΔPSD induced by DT). Notably, no statistically significant correlations were observed in the younger adult group; therefore, the results reported below are specific to the older adult group. In all reported models, the F-values represent the test statistic for the overall significance of the regression model, accompanied by their respective degrees of freedom. For the older adult group: MoCA-B scores showed strong positive correlations with Acc (*F*(1,16) = 45.981, *p*(*fdr*) < 0.001, *r* = 0.861, *R*^2^ = 0.742) and Perf (*F*(1,16) = 21.386, *p*(*fdr*) < 0.001, *r* = 0.756, *R*^2^ = 0.572), and a moderate negative correlation with T (*F*(1,16) = 9.309, *p*(*fdr*) = 0.014, *r* = −0.606, *R*^2^ = 0.368) (Figure 7A-Upper panel). Among regression models, the model based on DT Accuracy demonstrated the highest explanatory power. Additionally, ΔPSD exhibited strong positive correlations with Acc (*F*(1,16) = 30.435, *p*(*fdr*) = 0.003, *r* = 0.810, *R*^2^ = 0.655), moderate positive correlations with MoCA-B (*F*(1,16) = 19.367, *p*(*fdr*) < 0.001, *r* = 0.740, *R*^2^ = 0.548) and Perf (*F*(1,16) = 12.023, *p*(*fdr*) = 0.006, *r* = 0.655, *R*^2^ = 0.429), and a non-significant negative trend with T (*r* = −0.260, *p* > 0.05) (Figure 7A-Lower panel). The Acc-based ΔPSD model again showed optimal explanatory power. As shown in Figure 7B, regarding metabolic indicators, VFA showed significant negative correlations with both MoCA-B scores (*F*(1,16) = 6.364, *p*(*fdr*) = 0.032, *r* = −0.533, *R*^2^ = 0.285) and ΔPSD (*F*(1,16) = 5.588, *p*(*fdr*) = 0.047, *r* = −0.509, *R*^2^ = 0.259). Conversely, lower limb extensor peak torque was positively correlated with MoCA-B (*F*(1,16) = 5.586, *p*(*fdr*) = 0.036, *r* = 0.509, *R*^2^ = 0.259) and ΔPSD (*F*(1,16) = 10.393, *p*(*fdr*) = 0.010, *r* = 0.628, *R*^2^ = 0.394). No significant associations were found between SMI and either MoCA-B or ΔPSD. Detailed statistical results are shown in Table S3.

## 4. Discussion

In order to study the neuroimaging indicators of age-related cognitive decline under DT and elucidate the changing process of brain aging, we analyzed behavioral indicators, EEG neural oscillations, and connectivity changes in brain functional networks during DT in young and old groups, and explored the statistical correlations of various modal indicators, including metabolic indicators.

### 4.1. Task Performance

Intergroup comparisons revealed significant age effects: younger adults outperformed older adults in Acc and Perf, while older adults exhibited prolonged T. Reduced Acc in older adults suggests impaired attentional resource allocation during multitasking, consistent with reports of increased susceptibility to interference in aging populations [27]. Longer T values align with age-related declines in processing speed [28]. The superior Perf in younger adults reflects their preserved cognitive flexibility and efficient task management strategies [29,30], highlighting the differential impact of aging on distinct cognitive domains.

### 4.2. Power Spectral Density

Neural oscillation changes pre-to-post DT reveal age-related cognitive processing differences, with fundamental distinctions between younger and older adults during dual-task performance that elucidate neurophysiological mechanisms of cognitive decline.

Younger adults exhibited significantly enhanced power spectral density (PSD) in the Alpha frequency band across bilateral central, parietal, and temporal regions following dual-task completion, whereas older adults showed only focal Gamma-band enhancements at right parieto-occipital (PO4) and temporal (T6) sites. This restricted modulation is interpreted as a specific resource reallocation associated with high cognitive load tasks. The older adults’ results may reflect an attempt to compensate for reduced neural efficiency by enhancing visual (via PO4, a parieto-occipital site) and auditory (via T6, a temporal site) processing efficiency to maintain cognitive performance [31,32]. Conversely, the divergence in activated frequency bands and brain regions between the two groups indicates that older adults rely on localized, high-energy Gamma activation as a compensatory mechanism because they fail to recruit the extensive Alpha-band networks available to young controls. This lack of Alpha modulation (which typically supports efficient attentional resource allocation in younger adults [33,34,35]) forces the elderly to depend on spatially limited yet metabolically costly Gamma oscillations to cope with increased cognitive load.

These findings highlight fundamental differences in how younger and older brains adapt to cognitive demands, with younger adults demonstrating greater neural flexibility and older adults relying on compensatory focal activation.

### 4.3. Functional Connectivity

In addition to examining localized neural oscillations, we further investigated age-related differences in large-scale network interactions by comparing pre- and post-task resting-state functional connectivity. Younger adults showed enhanced post-task connectivity across multiple bands, with left temporal and right prefrontal hubs in Alpha/Beta networks (Figure 4), reflecting efficient attention (Alpha) and cognitive control (Beta) [8,36,37]. Network topology metrics provide important functional characterization of age-related neural reorganization. Older adults lacked connectivity changes, indicating impaired network adaptation. Topology analyses revealed younger adults’ increased efficiency (higher Eg, lower Lp) in Theta-Alpha-Beta bands (Figure 5), supporting flexible neural recruitment [38,39,40]. Nodal metrics showed younger adults’ frequency-specific reorganization: elevated Dc in temporal (Alpha) and central (Gamma) regions with reduced Bc, indicating optimized routing. Older adults displayed localized adaptations (e.g., Dc increases in temporal (Theta) and central (Beta) regions), suggesting compensatory attempts that did not translate to behavioral improvement [41]. The findings collectively suggest that age-related cognitive decline may stem from a progressive deterioration in the brain’s capacity for coordinated, frequency-specific network reorganization.

### 4.4. Correlation Analysis

Having characterized age-related differences in neural oscillations and functional connectivity, we further examined systematic relationships among metabolic indices, neural oscillations, and cognitive function in older adults to validate the dual-task paradigm’s assessment efficacy. Our analyses established critical multimodal interactions that provide novel insights into brain aging mechanisms. In older adults, MoCA-B scores strongly correlated with task Acc and Perf, and inversely with T. Notably, Acc explained 74.2% of MoCA-B variance. ΔPSD values positively correlated with Acc, MoCA-B, and Perf, suggesting neural adaptability reflects cognitive reserve [42,43]. Metabolic measures showed that visceral fat area (VFA) negatively correlated with MoCA-B and ΔPSD, while lower limb strength positively correlated with both (MoCA-B, ΔPSD). Limb strength explained 39.4% of ΔPSD variance, surpassing MoCA-B (25.9%), highlighting its preferential role in neural regulation. These findings align with emerging theories of metabolic brain aging. Specifically, the negative impact of visceral adiposity on cognition supports evidence that systemic metabolic dysfunction and adiposity drive neuroinflammation and cognitive decline [44,45]. Furthermore, the protective role of lower limb strength may reflect enhanced cerebral energy metabolism; recent studies highlight that maintaining neuronal function in aging relies on efficient astrocyte–neuron metabolic coupling and mitochondrial health, which can be preserved through physical interventions [46,47]. Collectively, this evidence reinforces current metabolic brain aging frameworks and underscores the critical role of lower limb strength in maintaining cognition [48,49,50].

Therefore, we propose that integrating lower limb strength training with visceral adiposity management may effectively preserve cognitive function by enhancing neural oscillation plasticity. This suggests multidimensional interventions (such as metabolic, physical, and cognitive) could shift geriatric care from treatment to prevention.

### 4.5. Limitations

While revealing novel age-related dual-task differences, several methodological considerations limit the generalizability of our findings. First, the sample size was relatively small, which may affect the statistical power and the robustness of the observed effects. Second, we did not control for potential confounding factors such as years of education, socioeconomic status, or access to healthcare. These variables are known to influence cognitive performance. Third, the generalizability of our findings to the “oldest-old” population (aged 80+) is constrained by the upper age limit of our cohort (70 years). This age ceiling was dictated by safety considerations inherent to the experimental protocol, which required sustained moderate-intensity cycling. Individuals aged 80 and above were systematically excluded during screening due to an inability to maintain the required physical intensity or the presence of comorbidities that posed unacceptable exercise risks. Finally, although EEG was recorded throughout, motion artifacts during cycling restricted analyses to pre/post-task resting-state data.

## 5. Conclusions

This study integrates behavioral, neural oscillatory, connectomic, and metabolic evidence to delineate the mechanisms of age-related cognitive decline under DT conditions. Our findings reveal that older adults exhibited reduced accuracy and slower processing speed alongside localized gamma-band enhancements, suggesting compensatory neural recruitment, whereas younger adults maintained integrated multi-band activation. Connectivity analyses revealed distinct age-related network strategies: younger adults exhibited enhanced hub connectivity in core regions with more direct information pathways, while older adults displayed less efficient connectivity patterns. The identified metabolic-cognitive-neural interactions position the DT paradigm as a sensitive tool for early cognitive vulnerability detection. These findings not only elucidate the mechanisms of age-related cognitive decline but also support the DT paradigm as a sensitive and efficient tool for assessing cognitive vulnerability in aging populations. The identified metabolic-cognitive-neural interactions further underscore the potential of combined metabolic-motor-cognitive interventions for proactive aging management.

## Figures and Tables

**Table 1:** Demographic characteristics of the older adult group and the younger adult group.

	Group O	Group Y	^2^ *U* or *t*	*p*
Gender (male/female)	9/9	10/9	*U* = 175.000	0.875
Age (years)	66.722 ± 6.086	23.105 ± 1.729	*t* = −30.010	<0.001
Years of Education (years)	10.111 ± 3.660	16.474 ± 1.349	*U* = 175.000	<0.001
Height (cm)	169.460 ± 7.601	170.460 ± 9.324	*t* = 0.300	0.767
BMI (kg/m^2^)	24.608 ± 4.185	23.769 ± 3.277	*t* = −0.297	0.769
SMI (kg/m^2^)	7.350 ± 0.578	6.924 ± 0.789	*t* = −1.866	0.070
VFA (cm^2^)	98.533 ± 42.518	83.642 ± 36.440	*t* = −1.146	0.260
^1^ P_LM (Nm)	109.781 ± 24.070	126.753 ± 46.731	*t* = 1.377	0.177

^1^ P_LM: Peak torque of lower extremity extensor muscles. ^2^ Values in the ‘*U* or *t*’ column denote the statistical test used: *U* values signify non-normal data distributions analyzed via the Mann–Whitney U test, while *t* values signify normal distributions analyzed via independent samples *t*-tests.

**Table 2:** Statistical results of PSD.

Group	Band	Electrode	*W* or *t*	*p*	^1^ *p*(*fdr*)	^2^ *d* or ^3^ $\hat{p}$	*Power*
O	Gamma	PO4	*W* = 179	<0.001	0.030	$\hat{p}$ = 0.936	90.0%
		T6	*W* = 179	<0.001	0.030	$\hat{p}$ = 0.935	90.0%
Y	Delta	PZ	*W* = 190	<0.001	0.015	$\hat{p}$ > 0.999	96.9%
		T6	*W* = 190	<0.001	0.015	$\hat{p}$ > 0.999	96.9%
	Theta	C4	*t* = 2.857	0.005	0.021	*d* = 0.655	77.1%
	Alpha	FZ	*W* = 169	0.003	0.025	$\hat{p}$ = 0.889	84.6%
		F3	*W* = 159	0.010	0.027	$\hat{p}$ = 0.867	73.1%
		FC1	*W* = 171	0.002	0.012	$\hat{p}$ = 0.900	86.4%
		FC2	*W* = 190	<0.001	0.003	$\hat{p}$ > 0.999	96.9%
		FC5	*W* = 162	0.007	0.047	$\hat{p}$ = 0.853	76.9%
		CZ	*W* = 164	0.005	0.026	$\hat{p}$ = 0.863	79.3%
		C3	*W* = 184	<0.001	0.005	$\hat{p}$ = 0.968	94.8%
		C4	*W* = 164	0.005	0.003	$\hat{p}$ = 0.863	79.3%
		T3	*W* = 165	0.005	0.027	$\hat{p}$ = 0.868	80.4%
		T4	*W* = 152	0.022	0.047	$\hat{p}$ = 0.800	63.1%
		CP5	*W* = 179	<0.001	0.008	$\hat{p}$ = 0.942	92.2%
		CP6	*t* = 2.094	0.025	0.047	*d* = 0.421	41.1%
		T5	*W* = 162	<0.001	0.036	$\hat{p}$ = 0.853	76.9%
		PO4	*W* = 148	0.033	0.038	$\hat{p}$ = 0.779	56.8%
	Beta	FC2	*W* = 177	<0.001	0.045	$\hat{p}$ = 0.932	91.0%
		T5	*W* = 173	0.002	0.045	$\hat{p}$ = 0.911	88.1%

^1^ *p*(*fdr*): FDR-corrected *p* value. ^2^ *d*: *Cohen’s d*. ^3^ $\hat{p}$: Estimated effect size in nonparametric power analysis.

## Data Availability

Anonymized data may be made available from the corresponding author upon reasonable request, subject to approval by the institutional ethics committee.

## Supplementary Materials

The following supporting information can be downloaded at: https://www.mdpi.com/article/10.3390/brainsci16030335/s1. Figure S1: Correspondence between electrodes and brain regions. Table S1: Detailed brain region-to-electrode correspondences. Table S2: Statistical results of the strength of brain network connections at the whole-brain level. Table S3: S3.1 Correlation Matrix: MoCA-B with ΔPSD, Dual-Task Performance (Acc, T, Perf) and (VFA, L_PM, SMI). S3.2 Correlation Matrix: ΔPSD with Metabolic indicators (SMI, VFA, L_PM) and Dual-Task Performance (Acc, T, Perf). Table S4: Statistical results of nodal degree centrality (Dc). Table S5: Statistical results of betweenness centrality (Bc). Wpli_compute.m.

## Abbreviations

The following abbreviations are used in this manuscript:

EEG	electroencephalography
MMSE	Mini-Mental State Examination
MoCA	Montreal Cognitive Assessment
MoCA-B	Montreal Cognitive Assessment—BeiJing
fMRI	functional Magnetic Resonance Imaging
PET	Positron Emission Tomography
DT	Dual Task
RHR	Resting Heart Rate
BMI	Body Mass Index
SMI	Muscle Mass Index
P_LM	Lower Limb Extensor Peak Torque
VFA	Visceral Fat Area
BIA	Bioelectrical Impedance Analysis
PSD	Power Spectral Density
wPLI	Weighted Phase Lag Index
Eg	Global Efficiency
Eloc	Local Efficiency
Lp	Characteristic Path Length
Cp	Clustering Coefficient
Dc	Degree Centrality
Bc	Betweenness Centrality
Acc	Accuracy Rate
T	Mean Response Time
Perf	Performance Index
NBS	Network Based Statistic
FDR	False Discovery Rate
