# Supplementary material for: Neural Oscillatory and Network Signatures of Age-Related Cognitive Decline Under Motor-Cognitive Dual-Task Conditions
Source: Brain Sci. 2026 Mar 21;16(3):335. doi: 10.3390/brainsci16030335 (PMC13024022; doi:10.3390/brainsci16030335)
Supplement: Supplementary file 1 [file brainsci-16-00335-s001.zip › Supplemental Materials_FigureS1.pdf]

Figure S1: Correspondence between electrodes and brain regions.

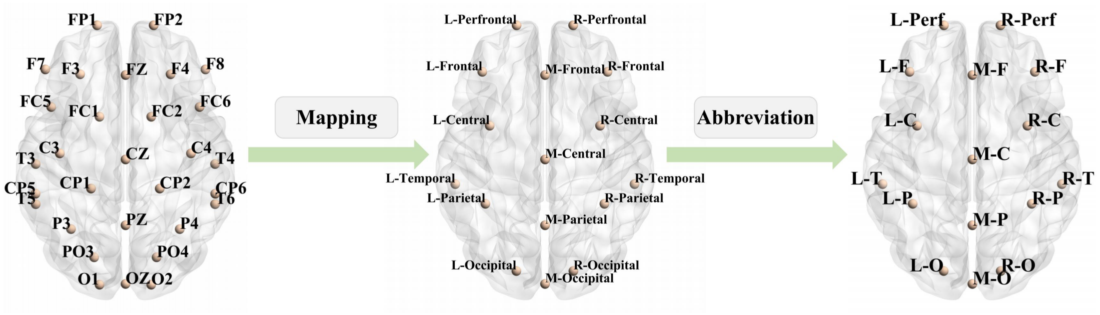

eFigure 1 Correspondence between electrodes and brain regions. The three brain topology plots (left to right) illustrate: (1) original electrode positions, (2) parcellated anatomical regions following atlas-based classification, and (3) abbreviatied region labels.
