# Supplementary material for: Neural Oscillatory and Network Signatures of Age-Related Cognitive Decline Under Motor-Cognitive Dual-Task Conditions
Source: Brain Sci. 2026 Mar 21;16(3):335. doi: 10.3390/brainsci16030335 (PMC13024022; doi:10.3390/brainsci16030335)
Supplement: Supplementary file 1 [file brainsci-16-00335-s001.zip › Supplemental Materials_TableS1.pdf]

Table S1: Detailed brain region-to-electrode correspondences.

Region	The corresponding electrode
Left Prefrontal	FP1
Right Prefrontal	FP2
Left Frontal	F3,F7
Right Frontal	F4,F8
Midline Frontal	FZ
Left Central	C3,FC1,FC5
Right Central	C4,FC2,FC6
Midline Central	CZ
Left Temporal	T3,T5
Right Temporal	T4,T6
Left Parietal	P3,CP1,CP5
Right Parietal	P4,CP2,CP6
Midline Parietal	PZ
Left Occipital	O1,PO3
Right Occipital	O2,PO4
Midline Occipital	OZ
