# Supplementary material for: Neural Oscillatory and Network Signatures of Age-Related Cognitive Decline Under Motor-Cognitive Dual-Task Conditions
Source: Brain Sci. 2026 Mar 21;16(3):335. doi: 10.3390/brainsci16030335 (PMC13024022; doi:10.3390/brainsci16030335)
Supplement: Supplementary file 1 [file brainsci-16-00335-s001.zip › Supplemental Materials_TableS2.pdf]

Table S2: Statistical results of the strength of brain network connections at the whole-brain level.

Group	Band	Mean ± SD (pre-)	Mean ± SD (post-)	*p*	*t*	*d*	*Power*
O	Delta	0.446±0.014	0.441±0.024	0.393	-0.877	0.207	12.9%
	Theta	0.456±0.028	0.467±0.045	0.450	0.774	0.182	11.0%
	Alpha	0.412±0.023	0.411±0.023	0.878	-0.156	0.037	3.5%
	Beta	0.240±0.017	0.243±0.017	0.651	0.461	0.109	6.4%
	Gamma	0.227±0.015	0.231±0.026	0.587	0.554	0.131	7.5%
Y	Delta	0.444±0.020	0.443±0.023	0.767	-0.301	0.069	4.7%
	Theta	0.442±0.023	0.454±0.020	0.023	2.485	0.570	65.2%
	Alpha	0.414±0.031	0.411±0.035	<0.001	4.186	0.960	97.7%
	Beta	0.239±0.015	0.252±0.019	0.005	3.229	0.741	86.3%
	Gamma	0.232±0.020	0.244±0.014	0.015	2.691	0.617	72.1%

d: Cohen's *d*.
