# Supplementary material for: Neural Oscillatory and Network Signatures of Age-Related Cognitive Decline Under Motor-Cognitive Dual-Task Conditions
Source: Brain Sci. 2026 Mar 21;16(3):335. doi: 10.3390/brainsci16030335 (PMC13024022; doi:10.3390/brainsci16030335)
Supplement: Supplementary file 1 [file brainsci-16-00335-s001.zip › Supplemental Materials_TableS3.pdf]

Table S3.1 Correlation Matrix: MoCA-B with ΔPSD, Dual-Task Performance (Acc, T, Perf) and (VFA, L_PM, SMI).

		Acc	T	Perf	ΔPSD	VFA	L_PM	SMI
MoCA-B	**Pearson (r)**	.861**	-.606**	.756**	.740**	-.533*	.509*	.491
	p	<.001	.008	<.001	<.001	.023	.031	.053
	***p*(fdr)**	<.001	.014	<.001	<.001	.032	.036	.053
	**95% CI (lower limit)**	.650	-.917	.489	.484	-.834	.527	.257
	**95% CI (upper limit)**	.961	-.146	.909	.928	-.078	.904	.876

Set the bootstrapping sample size to 2000.

p(fdr): FDR-corrected p value.

Table S3.2 Correlation Matrix: ΔPSD with Metabolic indicators (SMI, VFA, L_PM) and Dual-Task Performance (Acc, T, Perf) .

		VFA	L_PM	SMI	Acc	T	Perf
ΔPSD	Pearson (r)	-.509	.628**	.431	.810**	-.260	.655**
	p	.031	.005	.063	<.001	.125	.002
	p(fdr)	.076	.047	.010	.002	.125	.006
	95% CI (lower limit)	-.712	.698	.187	.489	-.788	.408
	95% CI (upper limit)	-.081	.911	.763	.899	.081	.856

Set the bootstrapping sample size to 2000.
p(fdr): FDR-corrected p value.
