# Supplementary material for: Neural Oscillatory and Network Signatures of Age-Related Cognitive Decline Under Motor-Cognitive Dual-Task Conditions
Source: Brain Sci. 2026 Mar 21;16(3):335. doi: 10.3390/brainsci16030335 (PMC13024022; doi:10.3390/brainsci16030335)
Supplement: Supplementary file 1 [file brainsci-16-00335-s001.zip › Supplemental Materials_TableS4.pdf]

Table S4: Statistical results of nodal degree centrality (Dc).

Group	Band	Region	***W* or *t***	***p***	***p*(fdr)**	***d* or $\hat{p}$**	***Power***
O	Theta	L-T	*t*=2.513	0.023	0.049	*d*=0.592	65.9%
	Beta	M-C	*t*=3.026	0.008	0.029	*d*=0.713	81.4%
Y	Delta	R-F	*t*=-2.884	0.011	0.025	*d*=0.662	77.9%
		L-O	*t*=-2.793	0.018	0.043	*d*=0.641	75.2%
	Alpha	R-C	*t*=3.583	0.002	0.013	*d*=0.822	92.3%
		M-C	*t*=2.658	0.021	0.047	*d*=0.610	71.0%
		L-T	*t*=3.588	0.002	0.011	*d*=0.823	92.4%
	Beta	L-F	*t*=-2.799	0.012	0.027	*d*=0.642	75.4%
	Gamma	L-C	*t*=2.713	0.018	0.045	*d*=0.622	72.8%

p(fdr): FDR-corrected *p* value.

d: Cohen's *d*.

$\hat{p}$: Estimated effect size in nonparametric power analysis.
