# Supplementary material for: Neural Oscillatory and Network Signatures of Age-Related Cognitive Decline Under Motor-Cognitive Dual-Task Conditions
Source: Brain Sci. 2026 Mar 21;16(3):335. doi: 10.3390/brainsci16030335 (PMC13024022; doi:10.3390/brainsci16030335)
Supplement: Supplementary file 1 [file brainsci-16-00335-s001.zip › Supplemental Materials_TableS5.pdf]

Table S5: Statistical results of betweenness centrality (Bc).

Group	Band	Region	***W* or *t***	***p***	***p*(fdr)**	***d* or $\hat{p}$**	***Power***
O	Delta	L-C	t=2.981	0.009	0.025	d=0.703	80.2%
	Theta	L-T	W=161	0.018	0.041	$\hat{p}$=0.942	90.8%
Y	Delta	L-F	t=2.723	0.015	0.031	d=0.625	73.1%
		L-O	W=171	0.002	0.012	$\hat{p}$>0.999	96.1%
	Alpha	L-PF	W=158	0.013	0.028	$\hat{p}$=0.924	88.4%
	Beta	L-F	t=-3.061	0.007	0.035	d=0.702	82.5%

p(fdr): FDR-corrected *p* value.

d: Cohen's *d*.

$\hat{p}$: Estimated effect size in nonparametric power analysis.
